# Supplementary figures and images for: Prevalence of type 2 diabetes mellitus and impaired fasting glucose, and their associated lifestyle factors among teachers in the CLUSTer cohort
Source: PeerJ. 2024 Jan 22;12:e16778. doi: 10.7717/peerj.16778 (PMC10809994; doi:10.7717/peerj.16778)

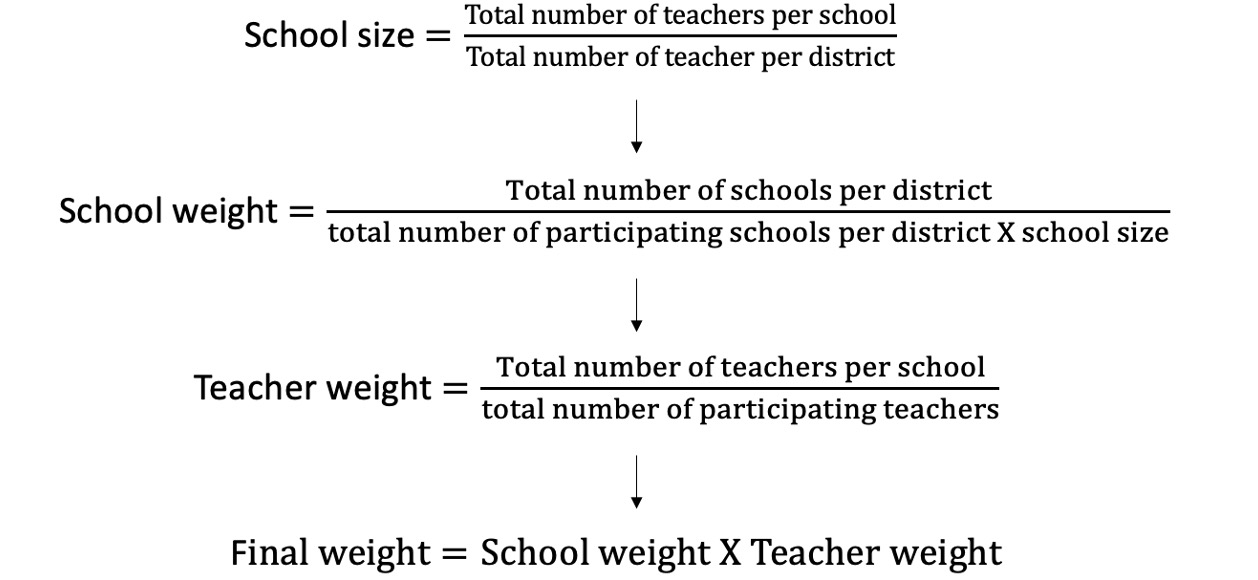

Supplement: Figure S1 [file peerj-12-16778-s001.jpg]

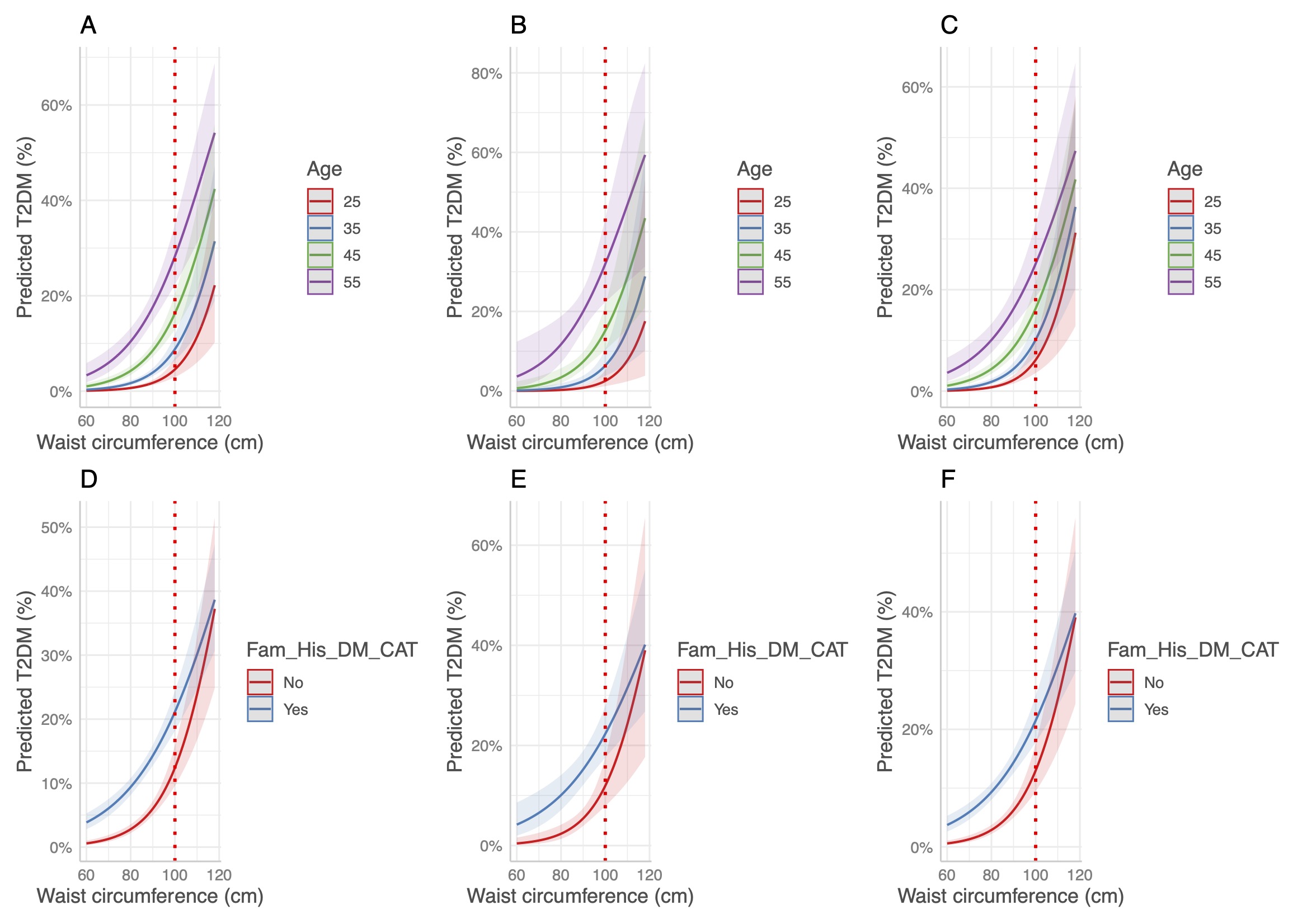

Supplement: Figure S2 — (A & D = Both male and female teachers), (B & E = Male teachers), (C & F = Female teachers) [file peerj-12-16778-s002.jpg]

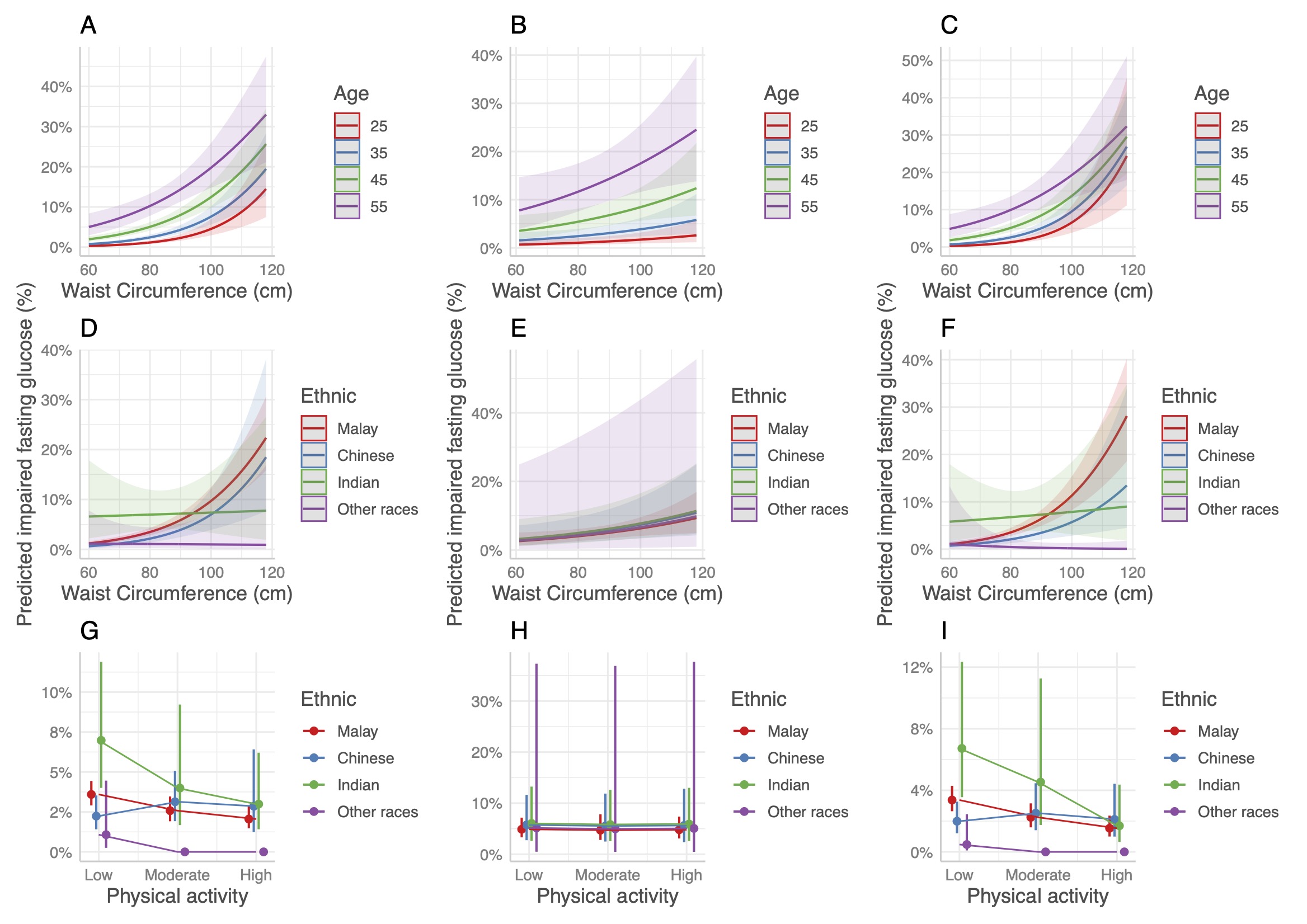

Supplement: Figure S3 — (A, D & G = Both male and female teachers), (B, E & H = Male teachers), (C, F & I = Female teachers). Noted that interaction terms were remove for regression model on male teachers due to errors while executing the R codes. [file peerj-12-16778-s003.jpg]
