# Supplementary material for: Prevalence of type 2 diabetes mellitus and impaired fasting glucose, and their associated lifestyle factors among teachers in the CLUSTer cohort
Source: PeerJ. 2024 Jan 22;12:e16778. doi: 10.7717/peerj.16778 (PMC10809994; doi:10.7717/peerj.16778)
Supplement: Table S1 [file peerj-12-16778-s004.docx]

**Supplemental Table S1. Operational definitions on variables**

| Component | Variable | Types | Operational definitions |
| --- | --- | --- | --- |
| Demographic | Age | Scale | Teachers’ age while serving in schools |
|  | Sex | Binary | Male/female |
|  | Ethnicity | Categorical | Malay/Chinese/Indian/Others |
|  | Marital | Categorical | Single/married/divorced/widowed |
|  | Education level | Categorical | Secondary/diploma/degree/>=master |
|  | Family history of T2DM | Binary | No/yes |
| Lifestyles | Fruit consumption | Scale | Servings/day |
|  | Vegetable consumption | Scale | Servings/day |
|  | Fruit and vegetable consumption | Binary | >=2 fruits and >=3 vegetables servings daily/no (Ahmad *et al.*, 2012; Nutritional Department Ministry of Health Malaysia., 2020) |
|  | Physical activity  Physical activity categorical | Scale  Categorical | Measured using International Physical Activity Questionnaire (short edition) (Craig *et al.*, 2017) (Cheng, 2016) |
|  | Duration of sitting | Scale | Minutes/day |
|  | Smoking status | Binary | No/yes |
|  | Alcohol consumption | Binary | No/yes |
|  | Waist circumference | Scale | Centimetre |
|  | Central obesity | Categorical | Male: (waist circumference >= 90cm)  Female: (waist circumference >= 80cm)  (Lear *et al.*, 2010) |
|  | Sleeping hours (weekday/weekend) | Scale | Hours/day |
|  | Anxiety/Depression/Stress | Scale | Measured using DASS-21 (Lovibond & Lovibond, 1996) |
| Outcomes | T2DM status | Binary | Known and undiagnosed T2DM  Known T2DM = clinically diagnosed  Undiagnosed T2DM = undiagnosed T2DM but with fasting blood glucose exceeded 6.1 mmol/L (Institute for Public Health, 2015) |
|  | IFG | Binary | No T2DM but with fasting blood glucose exceeded 5.6 mmol/L but lower than 6.1 mmol/L (Institute for Public Health, 2015) |

**References**

Ahmad, J., Wahab, S., Hamid, A., & Pardi, M. (2012). Malaysian Food Pyramid. Retrieved from http://www.myhealth.gov.my/en/malaysian-food-pyramid-2/

Cheng, H. L. (2016). *A simple, easy-to-use spreadsheet for automatic scoring of the International Physical Activity Questionnaire (IPAQ) Short Form*. Retrieved from https://www.researchgate.net/publication/310953872_A_simple_easy-to-use_spreadsheet_for_automatic_scoring_of_the_International_Physical_Activity_Questionnaire_IPAQ_Short_Form?channel=doi&linkId=583bbee208ae3a74b4a06f27&showFulltext=true

Craig, C., Marshall, A., Sjostrom, M., Bauman, A., Lee, P., Macfarlane, D., . . . Stewart, S. (2017). International Physical Activity Questionnaire-Short Form. Retrieved from https://youthrex.com/wp-content/uploads/2019/10/IPAQ-TM.pdf

Institute for Public Health. (2015). National Health and Morbidity Survey 2015 (NHMS 2015). In (Vol. 2): Ministry of Health Malaysia Kuala Lumpur. Retrieved from https://iku.gov.my/images/IKU/Document/REPORT/nhmsreport2015vol2.pdf

Lear, S. A., James, P. T., Ko, G. T., & Kumanyika, S. (2010). Appropriateness of waist circumference and waist-to-hip ratio cutoffs for different ethnic groups. *European Journal of Clinical Nutrition, 64*(1), 42-61. doi:10.1038/ejcn.2009.70

Lovibond, S. H., & Lovibond, P. F. (1996). *Manual for the depression anxiety stress scales*: Psychology Foundation of Australia.

Nutritional Department Ministry of Health Malaysia. (2020). *"Piramid Makanan Malaysia 2020 – Mendidik Rakyat Mengambil Makanan Dengan Betul"*. Retrieved from <https://nutrition.moh.gov.my/en/piramid-makanan-malaysia-2020-mendidik-rakyat-mengambil-makanan-dengan-betul/>
