# Supplementary material for: Prevalence of type 2 diabetes mellitus and impaired fasting glucose, and their associated lifestyle factors among teachers in the CLUSTer cohort
Source: PeerJ. 2024 Jan 22;12:e16778. doi: 10.7717/peerj.16778 (PMC10809994; doi:10.7717/peerj.16778)
Supplement: Table S2 [file peerj-12-16778-s005.docx]

| Factors | T2DM (known and undiagnosed) | | | IFG | | |
| --- | --- | --- | --- | --- | --- | --- |
|  | **OR** | **95% CI** | ***p value*** | **OR** | **95% CI** | ***p value*** |
| Age | 1.09 | 1.08, 1.10 | <0.001 | 1.08 | 1.06, 1.09 | <0.001 |
| Sex  Male (reference)  Female | 1.00  0.52 | -  0.43, 0.63 | <0.001 | 1.00  0.54 | -  0.42, 0.70 | <0.001 |
| Ethnic  Malay (reference) | 1.00 | - | <0.001 | 1.00 | - | 0.002 |
| Chinese | 0.49 | 0.35, 0.68 |  | 0.92 | 0.67, 1.27 |  |
| Indian | 1.35 | 0.99, 1.86 |  | 1.77 | 1.17, 2.67 |  |
| Other races | 1.10 | 0.45, 2.70 |  | 0.16 | 0.04, 0.67 |  |
| Education |  |  | 0.03 |  |  | 0.89 |
| Primary (reference)  Degree | 1.00  0.68 | -  0.45, 1.03 |  | 1.00  0.94 | -  0.51, 1.74 |  |
| Master and above | 0.87 | 0.56,1.35 |  | 0.89 | 0.46, 1.70 |  |
| Marital status |  |  | <0.001 |  |  | 0.012 |
| Single (reference)  Married | 1.00  3.32 | -  2.21,4.98 |  | 1.00  1.74 | -  1.15, 2.64 |  |
| Divorced/Widowed | 5.99 | 3.20, 11.2 |  | 2.54 | 1.26, 5.09 |  |
| Family history of DM  No (reference)  Yes | 2.78 | 2.29, 3.38 | <0.001 | 1.00  1.75 | -  1.39, 2.21 | <0.001 |
| Waist circumference (cm) | 1.07 | 1.06, 1.08 | <0.001 | 1.06 | 1.05, 1.06 | <0.001 |
| Duration of sitting (minutes) | 1.00 | 1.00, 1.00 | 0.03 | 1.00 | 1.00, 1.00 | 0.61 |
| Physical activity (Mets/day/week)  Low (reference) |  |  | 0.72 |  |  | 0.034 |
| Moderate | 0.92 | 0.74, 1.14 |  | 0.81 | 0.63, 1.06 |  |
| High | 0.95 | 0.76, 1.19 |  | 0.68 | 0.50, 0.92 |  |
| Smoking status  No (reference)  Yes | 1.00  0.99 | -  0.58, 1.69 | 0.97 | 1.00  2.17 | -  1.27, 3.70 | 0.004 |
| (continue) | | | | | | |
| Factors | **T2DM (known and undiagnosed)** | | | **IFG** | | |
|  | **OR** | **95% CI** | ***p value*** | **OR** | **95% CI** | ***p value*** |
| Alcohol consumption  No (reference)  Yes | 1.00  0.76 | -  0.40, 1.43 | 0.40 | 1.00  1.40 | -  0.83, 2.35 | 0.21 |
| Sleep duration (hours/day) | 0.80 | 0.72, 0.88 | <0.001 | 0.81 | 0.71, 0.93 | 0.002 |
| Fruit & Vegetable consumption (servings/day)  Inadequate (reference)  Adequate | 1.00  0.90 | -  0.58, 1.40 | 0.65 | 1.00  1.65 | -  0.98, 2.80 | 0.061 |
| Depression score | 0.99 | 0.97, 1.00 | 0.07 | 1.01 | 0.99, 1.02 | 0.57 |
| Anxiety score | 0.99 | 0.98, 1.00 | 0.20 | 1.00 | 0.98, 1.02 | 0.90 |
| Stress score | 0.98 | 0.96, 0.99 | 0.003 | 0.99 | 0.97, 1.01 | 0.21 |

**OR = Odds Ratio, CI = Confidence interval; MetS = Metabolic equivalent of task; Adequate = two servings of fruits and three servings of vegetables per day.**
