# Supplementary material for: Prevalence of type 2 diabetes mellitus and impaired fasting glucose, and their associated lifestyle factors among teachers in the CLUSTer cohort
Source: PeerJ. 2024 Jan 22;12:e16778. doi: 10.7717/peerj.16778 (PMC10809994; doi:10.7717/peerj.16778)
Supplement: Table S3 [file peerj-12-16778-s006.docx]

| Model | Lifestyle factors | T2DM (known and undiagnosed) | | | IFG | | |
| --- | --- | --- | --- | --- | --- | --- | --- |
|  |  | **aOR** | **95% CI** | ***p value*** | **aOR** | **95% CI** | ***p value*** |
| Full ^a,b^ | **Waist circumference (cm)** | 1.05 | 1.04, 1.06 | <0.001 | 1.04 | 1.03, 1.06 | <0.001 |
|  | **Duration of sitting (minutes)** | 1.00 | 1.00, 1.00 | 0.2 | 1.00 | 1.00, 1.00 | 0.9 |
|  | **Physical activity (Mets/day/week)** |  |  |  |  |  |  |
|  | Low (reference)  Moderate | 1.00  0.87 | -  0.69, 1.10 | -  0.2 | 1.00  0.77 | -  0.58, 1.01 | -  0.059 |
|  | High | 0.81 | 0.63, 1.05 | 0.11 | 0.58 | 0.42, 0.81 | 0.001 |
|  | **Smoking status**  No (reference)  Yes | 1.00  0.52 | -  0.32, 1.05 | -  0.073 | 1.00  1.52 | -  0.83, 2.78 | -  0.2 |
|  | **Alcohol consumption (Yes)**  No (reference)  Yes | 1.00  0.80 | -  0.40, 1.60 | -  0.5 | 1.00  1.27 | -  0.69, 2.33 | -  0.4 |
|  | **Sleep duration (hours/day)** | 0.96 | 0.85, 1.07 | 0.5 | 0.92 | 0.79, 1.06 | 0.2 |
|  | **Fruit & Vegetable (Adequate)**  Inadequate (reference)  Adequate | 1.00  0.80 | -  0.50, 1.30 | -  0.4 | 1.00  1.72 | -  0.99, 3.00 | -  0.056 |
|  | **Depression score** | 1.00 | 0.97, 1.03 | > 0.9 | 1.03 | 0.98, 1.07 | 0.059 |
|  | **Anxiety score** | 1.01 | 0.99, 1.07 | 0.4 | 1.01 | 0.98, 1.04 | 0.6 |
|  | **Stress score** | 0.98 | 0.95, 1.01 | 0.13 | 0.96 | 0.93, 1.00 | 0.029 |
| Final ^c,d^ | **Waist circumference (cm)** | 1.14 | 1.08, 1.20 | < 0.001 | 1.10 | 1.05, 1.15 | < 0.001 |
|  | **Physical activity**  Low (reference)  Moderate  High | - | - | - | 1.00  0.71  0.56 | -  0.52, 0.98  0.40, 0.80 | -  0.036  0.001 |
|  | **Waist circumference*Age**  **Waist circumference*Family history of T2DM (Yes)**  **Waist circumference*Ethnicity (Chinese)**  **Waist circumference*Ethnicity (Indian)**  **Waist circumference*Ethnicity (Others)**  **Physical activity (Moderate)*Ethnicity (Chinese)**  **Physical activity (Moderate)*Ethnicity (Indian)** | 1.00  0.97  -  -  -  -  - | 1.00, 1.00  0.95, 0.99  -  -  -  -  - | 0.024  0.002  -  -  -  -  - | 1.00  -  1.01  0.95  0.94  1.99  0.78 | 1.00, 1.00  -  0.98, 1.04  0.91, 0.99  0.88, 1.01  0.99, 3.99  0.29, 2.13 | 0.046  -  0.6  0.014  0.10  0.052  0.6 |

**(Continue)**

| Model | Lifestyle factors | T2DM (known and undiagnosed) | | | IFG | | |
| --- | --- | --- | --- | --- | --- | --- | --- |
|  |  | **aOR** | **95% CI** | ***p value*** | **aOR** | **95% CI** | ***p value*** |
| Final ^c,d^ | **Physical activity (Moderate)*Ethnicity (Others)**  **Physical activity (High)*Ethnicity (Chinese)**  **Physical activity (High)*Ethnicity (Indian)**  **Physical activity (High)*Ethnicity (Others)** | -  -  -  - | -  -  -  - | -  -  -  - | 0.00  2.28  0.73  0.00 | 0.00, 0.00  0.83, 6.30  0.28, 1.88  0.00, 0.00 | < 0.001  0.110  0.500  < 0.001 |

**aOR = Adjusted Odds Ratio; CI = Confidence Interval; * = Interaction.**

**^a^ (T2DM) =** **Adjusted for all lifestyle variables, age, sex, ethnicity, education level, marital status, and family history of T2DM**

**^b^ (IFG) = Adjusted for all lifestyle variables, age, sex, ethnicity, marital status, and family history of T2DM**

**^c^ (T2DM) = Adjusted for age, ethnicity, and family history of T2DM**

**^d^ (IFG) = Adjusted for age, ethnicity, and family history of T2DM, waist circumference, and physical activity.**
