# Supplementary material for: Prevalence of type 2 diabetes mellitus and impaired fasting glucose, and their associated lifestyle factors among teachers in the CLUSTer cohort
Source: PeerJ. 2024 Jan 22;12:e16778. doi: 10.7717/peerj.16778 (PMC10809994; doi:10.7717/peerj.16778)
Supplement: Table S4 [file peerj-12-16778-s007.docx]

**Supplementary Table S4. Weighted descriptive statistical analysis on variables by gender**

| **Variables** | **n** | **Female**, n = 11,460^1^ | **Male**, n = 2,684^1^ |
| --- | --- | --- | --- |
| **Age** | 14,143 | 39.91±8.66 | 41.75±9.60 |
| **Ethnic** | 14,144 |  |  |
| Chinese |  | 15.0 (14.0, 16.0) | 11.0 (9.0, 12.0) |
| Indian |  | 8.0 (7.3, 8.8) | 8.1 (6.6, 10.0) |
| Malay |  | 76.0 (75.0, 77.0) | 81.0 (78.0, 83.0) |
| Other races |  | 0.9 (0.6, 1.2) | 0.6 (0.3, 1.2) |
| **Education** | 11,820 |  |  |
| Degree |  | 80.0 (79.0, 81.0) | 73.0 (71.0, 76.0) |
| Diploma |  | 2.5 (2.1, 3.0) | 2.8 (2.0, 3.9) |
| Master and above |  | 15 (14, 16) | 19 (17, 21) |
| Secondary |  | 3.0 (2.6, 3.5) | 4.7 (3.7, 6.1) |
| **Marital status** | 12,160 |  |  |
| Divorced |  | 1.6 (1.3, 2.0) | 0.4 (0.2, 1.0) |
| Married |  | 85.0 (84.0, 86.0) | 88.0 (86.0, 90.0) |
| Single |  | 12.0 (11.0, 13.0) | 10.0 (8.8, 12.0) |
| Widowed |  | 1.0 (0.8, 1.3) | 1.1 (0.6, 2.0) |
| **Family history of T2DM** | 11,834 | 52.0 (50.0, 53.0) | 48.0 (45.0, 52.0) |
| **Fruit consumption (servings/day)** | 8,971 | 0.89±1.01 | 0.79±1.01 |
| **Vege consumption (servings/day)** | 8,517 | 1.48±1.33 | 1.45±1.57 |
| **Fruit and Vegetable consumption** | 8,345 |  |  |
| Adequate |  | 3.9 (3.3, 4.5) | 3.7 (2.7, 5.0) |
| **Sleep hours (weekday)** | 11,214 | 5.96±1.01 | 5.87±0.95 |
| **Sleep hours (weekend)** | 11,261 | 6.70±1.11 | 6.40±1.20 |
| **Physical Activity (METs-minutes/week)** | 14,144 | 1,272.00±2,988.70 | 1,709.00±2,434.69 |
| **Duration of sitting (minutes)** | 5,637 | 191.96±149.00 | 190.34±146.64 |
| **Smoking status** | 11,724 | 0.4 (0.29, 0.65) | 24.0 (21.0, 27.0) |
| **Alcohol consumption** | 11,097 | 3.2 (2.8, 3.7) | 6.3 (5.1, 7.7) |
| **Waist circumference (cm)** | 13,548 | 79.88 (10.63) | 89.80 (10.98) |
| Abdominal obese |  | 53.0 (51.0, 54.0) | 50.0 (47.0, 53.0) |
| **Depression score** | 11,816 | 6.32±6.21 | 5.65±6.26 |
| **Anxiety score** | 11,844 | 8.44±6.43 | 7.78±6.53 |
| **Stress score** | 11,685 | 9.99±6.86 | 8.80±6.98 |
| **T2DM status** | 14,144 |  |  |
| Known T2DM |  | 3.5 (3.1, 4.0) | 6.8 (5.8, 8.1) |
| Undiagnosed T2DM |  | 4.8 (4.3, 5.4) | 8.6 (7.3, 10.0) |
| **Impaired Fasting glucose** | 12,757 |  |  |
| Yes |  | 5.1 (4.5, 5.7) | 8.6 (7.2, 10.0) |

**^1^ = mean ± SD or % (95% CI); SD = Standard deviation; CI = Confidence Interval.**
