# Supplementary material for: Prevalence of type 2 diabetes mellitus and impaired fasting glucose, and their associated lifestyle factors among teachers in the CLUSTer cohort
Source: PeerJ. 2024 Jan 22;12:e16778. doi: 10.7717/peerj.16778 (PMC10809994; doi:10.7717/peerj.16778)
Supplement: Table S5 [file peerj-12-16778-s008.docx]

**Supplemental Table S5. Comparison of multivariable regression model for prediction T2DM and IFG among teachers**

| Outcomes | Models | Akaike information criterion (AIC) | *p*-value |
| --- | --- | --- | --- |
| **T2DM** | **Original model (Figure 2)** | 5806.256 | 0.020 |
|  | **Model (inclusion of BMI)** | 5799.421 |  |
| **IFG** | **Original model (Figure 3)** | 4044.843 | < 0.001 |
|  | **Model (inclusion of BMI)** | 4025.276 |  |
