# Supplementary material for: Prevalence of type 2 diabetes mellitus and impaired fasting glucose, and their associated lifestyle factors among teachers in the CLUSTer cohort
Source: PeerJ. 2024 Jan 22;12:e16778. doi: 10.7717/peerj.16778 (PMC10809994; doi:10.7717/peerj.16778)
Supplement: Table S6 [file peerj-12-16778-s009.docx]

**Supplemental Table S6. Lifestyle factors associated with waist circumference among teachers in CLUSTer cohort**

| Factors | Adjusted Beta | 95% CI | *p*-value | GVIF | Adjusted GVIF |
| --- | --- | --- | --- | --- | --- |
| Physical activity |  |  | 0.034 | 1.2 | 1.0 |
| Low (reference) | - | - |  |  |  |
| Moderate | 0.51 | -0.21, 1.20 |  |  |  |
| High | -0.55 | -1.20, 0.13 |  |  |  |
| Sleep duration (hours) | -0.47 | -0.89, -0.06 | 0.025 | 1.6 | 1.3 |
| Anxiety | 0.05 | 0.01, 0.09 | 0.016 | 1.0 | 1.0 |

**Beta = Regression coefficient, CI = Confidence interval, GVIF = Generalised variance inflation factor.**

**Note: Lifestyle factors were adjusted among themselves together with age, sex, ethnicity, marital status, and family history of T2DM.**
