# Supplementary material for: Prevalence of type 2 diabetes mellitus and impaired fasting glucose, and their associated lifestyle factors among teachers in the CLUSTer cohort
Source: PeerJ. 2024 Jan 22;12:e16778. doi: 10.7717/peerj.16778 (PMC10809994; doi:10.7717/peerj.16778)
Supplement: Supplemental Information 15 [file peerj-12-16778-s015.docx]

**STROBE Statement—checklist of items that should be included in reports of observational studies**

|  | Item No. | Recommendation | Page  No.^#^ | Relevant information from manuscript | |
| --- | --- | --- | --- | --- | --- |
| **Title and abstract** | 1 | (*a*) Indicate the study’s design with a commonly used term in the title or the abstract | 4 | This study design was described in the abstract under the method section (line 23). | |
|  |  | (*b*) Provide in the abstract an informative and balanced summary of what was done and what was found | 4 | This information can be retrieved from lines 23–38. | |
| Introduction | | | | |  |
| Background/rationale | 2 | Explain the scientific background and rationale for the investigation being reported | 5 & 6 | Scientific background (lines 68-83)  Rationale (lines 84-87). | |
| Objectives | 3 | State specific objectives, including any prespecified hypotheses | 6 | Study objectives (lines 88-91). | |
| Methods | | | | |  |
| Study design | 4 | Present key elements of study design early in the paper | 4 | This study design was described in the abstract under the method section (line 23). | |
| Setting | 5 | Describe the setting, locations, and relevant dates, including periods of recruitment, exposure, follow-up, and data collection | 6 | Description of the details on study settings, dates, and other relevant information (lines 95-107). | |
| Participants | 6 | (*a*) *Cohort study*—Give the eligibility criteria, and the sources and methods of selection of participants. Describe methods of follow-up  *Case-control study*—Give the eligibility criteria, and the sources and methods of case ascertainment and control selection. Give the rationale for the choice of cases and controls  *Cross-sectional study*—Give the eligibility criteria, and the sources and methods of selection of participants | 6 | Eligible teachers who were free from psychiatric illness (lines 102-104). | |
|  |  | (*b*) *Cohort study*—For matched studies, give matching criteria and number of exposed and unexposed  *Case-control study*—For matched studies, give matching criteria and the number of controls per case | - | Not relevant. This is a cross-sectional study from the teacher CLUSTer cohort. | |
| Continued on next page |  |  |  |  | |
| Variables | 7 | Clearly define all outcomes, exposures, predictors, potential confounders, and effect modifiers. Give diagnostic criteria, if applicable | 6 | Description of study variables (lines 108-114). The details of each variable can be retrieved from Table S1. | |
| Data sources/ measurement | 8* | For each variable of interest, give sources of data and details of methods of assessment (measurement). Describe comparability of assessment methods if there is more than one group | Table S1 | The details of each variable can be retrieved from Table S1. | |
| Bias | 9 | Describe any efforts to address potential sources of bias | 6 | A multi-staged sampling strategy was adopted to reduce sampling bias among teachers (lines 97-100). | |
| Study size | 10 | Explain how the study size was arrived at | - | A minimum of 8,067 teachers needs to be recruited, according to Moy *et al*. (2014). Therefore, the authors considered adequate statistical power to carry out the analysis with the current number of recruited participants (n=14,144). | |
| Quantitative variables | 11 | Explain how quantitative variables were handled in the analyses. If applicable, describe which groupings were chosen and why | 6 &  Table S1 | Most quantitative variables were included in analyses without any transformation. They were described as a weighted mean with a standard deviation (lines 118-119). Additional variables (e.g., Fruit and vegetable consumption and central obesity) were computed from relevant continuous variables. The details can be found in Table S1. | |
| Statistical  methods | 12 | (*a*) Describe all statistical methods, including those used to control for confounding | 6 & 7 | All details on statistical analyses, both descriptive and inferential analyses, were elaborated (lines 115-135). | |
|  |  | (b) Describe any methods used to examine subgroups and interactions | 7 | Additional analysis on interaction (lines 133 – 134). | |
| Continued on next page |  |  |  |  | |

| Statistical  methods | 12 | (*c*) Explain how missing data were addressed | 7 | Missing values were imputed using the multiple imputation chained equation (MICE) (lines 125-126). |
| --- | --- | --- | --- | --- |
|  |  | (*d*) *Cross-sectional study*—If applicable, describe analytical methods taking account of sampling strategy | 6 | Complex analysis with an assigned weight (lines 117-121). |
|  |  | (*e*) Describe any sensitivity analyses | 11 &  Table S4 | An ad hoc rather than sensitivity analysis was done and described (lines 316-319) (Table S4). |
| Results | | | | |
| Participants | 13* | (a) Report numbers of individuals at each stage of study—eg numbers potentially eligible, examined for eligibility, confirmed eligible, included in the study, completing follow-up, and analysed | 7 & 8 | Line 143 reported on the number of participants involved. In addition, only teachers who managed to answer most survey questionnaires were included in the downstream inferential analysis (lines 180-181). |
|  |  | (b) Give reasons for non-participation at each stage | - | Participants who failed to answer most of the questionnaires were not included in downstream inferential analysis as they could potentially cause estimation biases. |
|  |  | (c) Consider use of a flow diagram | - | There were no complicated changes in participant numbers in this study. The details on the inclusion of participants in descriptive (line 143) and downstream inferential analysis were written in text (lines 180-181). Therefore, the authors did not present a flow diagram. |
|  |  |  |  |  |
| Continued on next page | | | | |
| Descriptive data | 14* | (a) Give characteristics of study participants (eg demographic, clinical, social) and information on exposures and potential confounders | Table 1 | The characteristics of study participants were presented in Table 1. |
|  |  | (b) Indicate number of participants with missing data for each variable of interest | Table 1 | The number of participants with missing data for each variable of interest was presented in Table 1. |
|  |  | (c) *Cohort study*—Summarise follow-up time (eg, average and total amount) | - | Not relevant |
| Outcome data | 15* | *Cohort study*—Report numbers of outcome events or summary measures over time | - | Not relevant |
|  |  | *Case-control study—*Report numbers in each exposure category, or summary measures of exposure | - | Not relevant |
|  |  | *Cross-sectional study—*Report numbers of outcome events or summary measures | 7 & 8 | All weighted prevalence’s of outcomes were presented in lines 162-165. |
| Main results | 16 | (*a*) Give unadjusted estimates and, if applicable, confounder-adjusted estimates and their precision (eg, 95% confidence interval). Make clear which confounders were adjusted for and why they were included | 8 & 9 ,Table S2, and S3 | The details on unadjusted estimates and adjusted estimates with 95% CI were displayed in Tables S2 and S3. Descriptions in texts in lines 182-245. |
|  |  | (*b*) Report category boundaries when continuous variables were categorized | Table S1 | The details on categorising fruit and vegetable consumption and central obesity status were described in Table S1. |
|  |  | (*c*) If relevant, consider translating estimates of relative risk into absolute risk for a meaningful time period | - | Not relevant |
| Other analyses | 17 | Report other analyses done—eg analyses of subgroups and interactions, and sensitivity analyses | 8 & 9 | The interaction between lifestyle factors and covariates was described separately on T2DM (lines 199-209) and IFG (lines 226-245). |
| Continued on next page | | | | |

| Discussion | | | | |
| --- | --- | --- | --- | --- |
| Key results | 18 | Summarise key results with reference to study objectives | 10-11 | Prevalence of T2DM and IFG (lines 252-277)  Lifestyles associated with T2DM and IFG (lines 278-324) |
| Limitations | 19 | Discuss limitations of the study, taking into account sources of potential bias or imprecision. Discuss both direction and magnitude of any potential bias | 12 | The study's limitations were highlighted in lines 328-334. |
| Interpretation | 20 | Give a cautious overall interpretation of results considering objectives, limitations, multiplicity of analyses, results from similar studies, and other relevant evidence | 12 | With a comparison of current findings with other relevant studies and study limitations throughout the discussion section (lines 249-334), the authors gave a statement to further strengthen the study in lines 335-341. |
| Generalisability | 21 | Discuss the generalisability (external validity) of the study results | - | Although no external data was collected, this study utilised a combination of multi-staged sampling and complex analysis with weights. Hence, the outcome is likely to be generalised to the teacher population in this country. |
| Other information | |  | | |
| Funding | 22 | Give the source of funding and the role of the funders for the present study and, if applicable, for the original study on which the present article is based | 12 | Acknowledgements to the funders (lines 351-355). |

**^#^** Those reference page numbers are based on the review Pdf generated from PeerJ submission platform.

*Give information separately for cases and controls in case-control studies and, if applicable, for exposed and unexposed groups in cohort and cross-sectional studies.
